# Australian women's judgements about using artificial intelligence to read mammograms in breast cancer screening

**DOI:** 10.1177/20552076231191057

**Published:** 2023-08-07

**Authors:** Stacy M Carter, Lucy Carolan, Yves Saint James Aquino, Helen Frazer, Wendy A Rogers, Julie Hall, Chris Degeling, Annette Braunack-Mayer, Nehmat Houssami

**Affiliations:** 1 Australian Centre for Health Engagement, Evidence and Values (ACHEEV), School of Health & Society, University of Wollongong, Wollongong, NSW, Australia; 2 St Vincent's Hospital BreastScreen, BreastScreen Victoria, Fitzroy, Victoria, Australia; 3 Philosophy Department and School of Medicine, Macquarie University, Sydney, NSW, Australia; 4 Daffodil Centre, University of Sydney, Joint Venture with Cancer Council NSW, Sydney, NSW, Australia; 5 Sydney School of Public Health, Faculty of Medicine and Health, University of Sydney, Sydney, NSW, Australia

**Keywords:** Artificial intelligence, mammography, qualitative research, bioethics, values, public health

## Abstract

**Objective:**

Mammographic screening for breast cancer is an early use case for artificial intelligence (AI) in healthcare. This is an active area of research, mostly focused on the development and evaluation of individual algorithms. A growing normative literature argues that AI systems should reflect human values, but it is unclear what this requires in specific AI implementation scenarios. Our objective was to understand women's values regarding the use of AI to read mammograms in breast cancer screening.

**Methods:**

We ran eight online discussion groups with a total of 50 women, focused on their expectations and normative judgements regarding the use of AI in breast screening.

**Results:**

Although women were positive about the potential of breast screening AI, they argued strongly that humans must remain as central actors in breast screening systems and consistently expressed high expectations of the performance of breast screening AI. Women expected clear lines of responsibility for decision-making, to be able to contest decisions, and for AI to perform equally well for all programme participants. Women often imagined both that AI might replace radiographers and that AI implementation might allow more women to be screened: screening programmes will need to communicate carefully about these issues.

**Conclusions:**

To meet women's expectations, screening programmes should delay implementation until there is strong evidence that the use of AI systems improves screening performance, should ensure that human expertise and responsibility remain central in screening programmes, and should avoid using AI in ways that exacerbate inequities.

## Introduction

This paper focuses on the use of artificial intelligence (AI) to read mammograms in breast cancer screening programmes. Defining AI is challenging: one general definition is ‘a collection of interrelated technologies used to solve problems that would otherwise require human cognition’.^1^ Contemporary AI systems for mammographic screen-reading are data-driven, generally employing convolutional neural networks or other deep learning technologies. AI, especially for disease detection, is at an early research and development stage in healthcare. As of July 2023,^2^ only 8.8% of 62,811 published healthcare AI studies had reached comparative or real-world evaluation.

There are ethical risks arising from healthcare AI adoption, including lack of evidence on clinical outcomes, algorithmic bias, automation bias, the risk of clinical de-skilling, potential to undermine patient choice and contestability of decisions, and potential implications for trust in healthcare services.^3^ It is often argued that responsible, accountable and trustworthy AI will be responsive to human values.^4,5^ However the relevant values or principles are often expressed at an abstract level. This makes it challenging to interpret general values and principles for particular AI use cases.

In a healthcare context, AI ethics must also connect with healthcare ethics and the expectation that there will be public and patient involvement (PPI) in healthcare design and delivery. This is reflected in recent calls for greater PPI in healthcare AI development and implementation.^6,7^ Reading mammograms in breast cancer screening programmes is a relatively advanced AI use case.^3^ In Australia, women aged 50–74 are offered publicly funded breast screening every 2 years via the national BreastScreen program; women aged 40–49 can access screening. Breast cancer screening has a strong PPI imperative due to its social and political significance. This suggests a need for engagement with publics—including women of screening age—about the design and implementation of breast screening AI and automated decision-making (ADM).

A few studies have engaged with women about breast screening AI in the UK and Europe.^8–10^ These suggest the majority of women prefer human to AI screen-readers,^8^ have a strong preference that AI decisions be checked by a human, and have only moderate support for the use of AI as a second reader.^9^ A British study reported positive or neutral views towards AI, with efficiency, reliability and safety perceived as positive attributes of AI systems. Hopes included that AI might release staff for higher-value activities, save money and address workforce shortages; concerns included preserving a ‘human touch’, avoiding discriminatory bias and privacy and uncertainty about governance.^10^ An Italian survey-based study reported positive views, but only if AI supported, rather than replaced, radiologists; women thought responsibility for errors should rest both with radiologists and with AI developers.^11^ We were unable to identify any in-depth qualitative studies exploring women's views on using AI in breast screening, and no qualitative or quantitative studies from Australia.

To help inform the implementation of AI in breast screening, and to add to the small existing international literature, we conducted a dialogue group-based study with women of breast screening age. The study objective was to understand women's values regarding the use of AI to read mammograms in breast cancer screening. The questions guiding this study were:

1. How do women respond to the idea of using AI to read mammograms in breast screening programmes?
2. How do women explain their judgements about the potential use of AI in breast screening?
3. How do women respond to the range of potentially competing values that could guide implementation of AI in breast screening?

## Methods

### Ethics approval

Ethics approval for the project was provided by the Human Research Ethics Committees of the University of Wollongong [2021/067].

### Approach

We employed dialogue groups, a method that encourages conversations about the ethical dimensions of technologies.^12,13^ Groups were run online on Zoom, from June to August 2021, with facilitation by senior researchers (SMC and YSJA) and support from assistant moderators (LC and JH), each group lasted approximately 2 hours.

### Participant recruitment and selection

We aimed to recruit eight groups of seven participants through an independent recruitment service (Taverner Research). Recruitment was from the general community using social media posts (Facebook) and via random digit dialling to households across Australia. All participants were in the age range for invitation to the public breast cancer screening program: 50–74 years old. For each group, we aimed for diversity in age, rural/regional/urban area of residence, educational qualifications and cultural background. Cultural and linguistic diversity was determined using two screening questions: (1) What is your country of birth? and (2) Do you identify with a culture other than the culture of your country of birth? Any respondent who identified a culture or country where English is not the first language was coded as being from a diverse group; we aimed to include two women from diverse backgrounds in each group to create a more supportive environment. The questions used by the recruitment agency to collect demographics are in Appendix 1 (see online supplementary material) Recruitment Screener Questionnaire.

Women who had been diagnosed with breast cancer more than 3 years prior to the research were included, but participated in two discrete groups of women with the same experience, to ensure a supportive environment. We excluded women diagnosed with breast cancer in the previous 3 years or in active treatment for breast cancer to minimise risk of causing distress and to ensure a focus on population screening for well women. We excluded women who could not speak English because we could not offer simultaneous translation. We excluded women who had worked in breast cancer care/screening in the last 5 years because they have more expert knowledge than the general population of women. All potential participants were sent a Participant Information Sheet (see Appendix 2 in online supplementary material) with comprehensive study details. Potential participants were contacted by research assistant (LC) before the group to invite any questions, record verbal consent to participate, and complete a short survey. Participants were also offered support to use Zoom. They received an $AUD100 retail voucher as compensation for their time.

### Data collection

Prior to the start of the dialogue groups, and again at the end of each group, women were asked to complete a short online survey of their knowledge, attitudes and intentions in relation to AI in breast screening (see Appendix 3 Survey Instrument in online supplementary material). Survey questions were replicated from our previous studies^14^ and others’ studies;^15^ all questions had been piloted in our previous studies.

**Figure 1.**
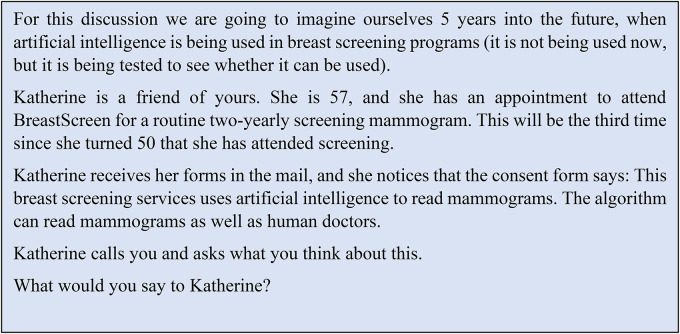
AI and breast screening scenario.

**Figure 2.**
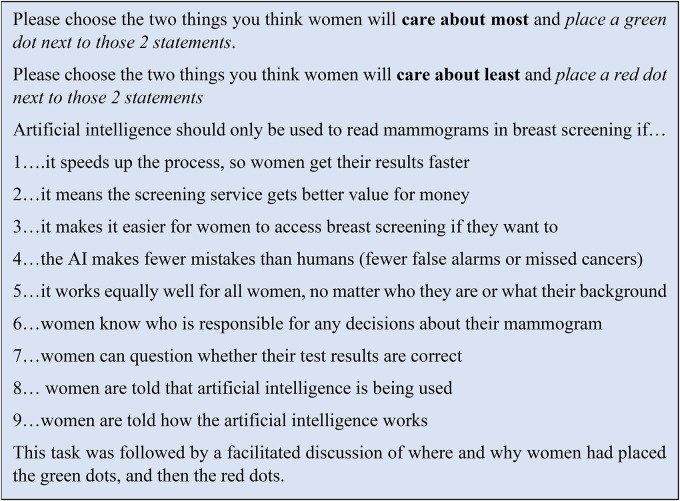
AI and breast screening scenario—value statements.

Dialogue groups started with a video and slide presentation outlining background on breast screening and AI (see Appendix 4 Background AI in Breast Screening in online supplementary material). The discussion commenced with a hypothetical scenario to explore initial reactions (Figure 1). We then introduced extension tasks, including a task focused on the aspects of breast screening AI that women would care about most and least (Figure 2). At the end of each group, women were asked what they would say about breast screening AI if they had an opportunity to speak to someone from BreastScreen. All tasks were developed in collaboration between the researchers and content experts, including ethicists with an interest in AI, and managers within Australian BreastScreen programmes. Discussions were audio-recorded and transcribed verbatim by a professional transcription service. All names were changed to pseudonyms before analysis commenced.

### Analysis

Demographic information was collected by the recruitment company, Taverner (see Appendix 1 in online supplementary material). We analysed the data with a focus on what women think should or should not happen regarding the use of AI in breast cancer screening, using methods from framework analysis.^16^ A memo was written about each group based on both the coded transcript and the field notes to summarise group dynamics, key insights arising from each group and comparisons between groups. Two researchers (SMC and LC) coded the first three transcripts, informed by the AI ethics, bioethics and screening literature. This coding was used to develop a coding structure grouped into higher-level concepts, with a focus on ethical issues and judgements. All transcripts were analysed using this structure by one researcher (LC), with regular input from SMC. The coding structure was adjusted for any additional concepts derived during further coding. When all transcripts were coded, LC and SMC sorted the concepts into core categories, which included initial responses, AI performance and evidence, human/AI roles, workflows and reasons for value judgements. A framework was constructed with one row for each group and one column for each of the core categories, to summarise and allow direct comparison of the talk of each group about the categories and their subcategories.

## Results/findings

### Participant characteristics

Participant characteristics are shown in Table 1. We aimed for seven women per group; however, attrition after recruitment reduced the size of some groups. Participants were broadly diverse, with a good representation of residential locations (urban, regional and rural). Methods to identify culturally and linguistically diverse respondents vary: approximately 34% of respondents were from culturally and linguistically diverse backgrounds as operationalised via our methods. Approximately 27.6% of Australians are born overseas (including in countries where English is the dominant language); approximately 22.8% use a language other than English at home.^17^ Our sample may thus have slightly over-represented culturally and linguistically diverse women. Our sample was skewed to higher levels of education: Australian women in this age group are 49% school educated (24% of our sample), 27% have a vocational qualification (22% of our sample) and 24% have a degree (54% of our sample).^18^

**Table 1.** Participant characteristics in each group.

	1	2	3	4	5	6	7	8	Total
	*n* = 6	*n* = 7	*n* = 6	*n* = 6	*n* = 5	*n* = 6	*n* = 7	*n* = 7	*n* = 50
Age of participants									
50–59	2	3	3	4	2	1	2	3	20
60–69	3	2	2	2	2	4	3	3	21
70–74	1	2	1	–	1	1	2	1	9
Personal history of breast cancer									
Yes	–	–	6	–	–	6	–	–	12
No	6	7	–	6	5	–	7	7	38
Educational/vocational qualifications									
High school	3	–	3	1	1	2	1	2	13
Trade certificate	–	4	1	1	1	1	1	2	11
University	3	3	2	4	3	3	5	3	26
Residential location									
Urban	2	1	2	3	3	3	3	3	20
Regional	3	3	3	2	1	1	3	3	19
Rural	1	3	1	1	1	2	1	1	11
Culturally/linguistically diverse									
Yes	2	2	2	2	2	2	3	2	17
No	4	5	4	4	3	4	4	5	33

### Survey responses

**Figure 3.**
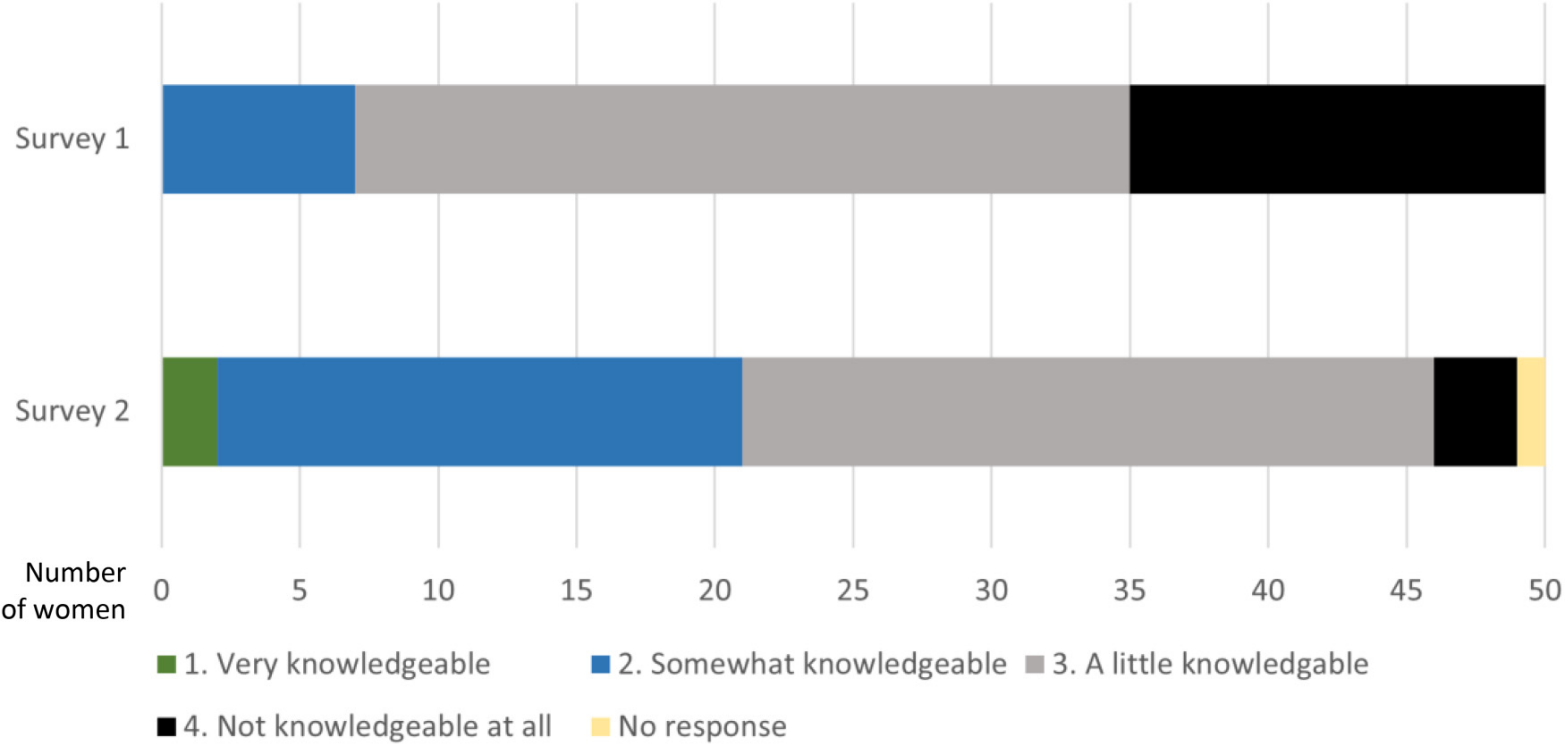
Pre-group discussion survey (Survey 1) and post-group discussion survey (Survey 2) responses: perceived knowledge about AI.

**Figure 4.**
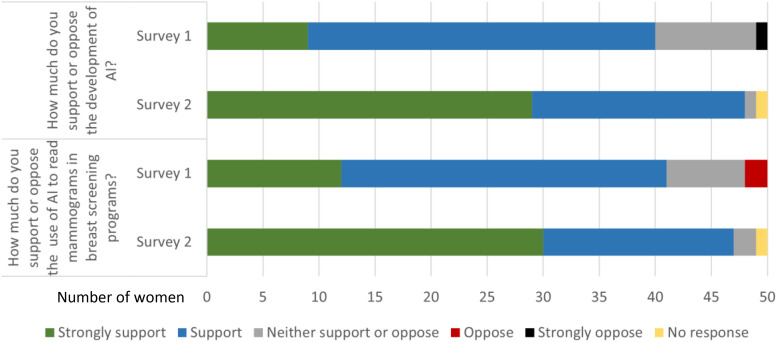
Pre-group discussion survey (Survey 1) and post-group discussion survey (Survey 2) responses: support for AI and for breast screening AI (note small sample size).

The post-group discussion survey (Survey 2) showed that women reported feeling more knowledgeable about AI as a result of participation, compared to their knowledge levels reported in Survey 1. Women expressed an increase both in support for the development of AI in general and in support for the use of AI in breast screening in particular in Survey 2 compared to Survey 1 (Figures 3 and 4).

**Figure 5.**
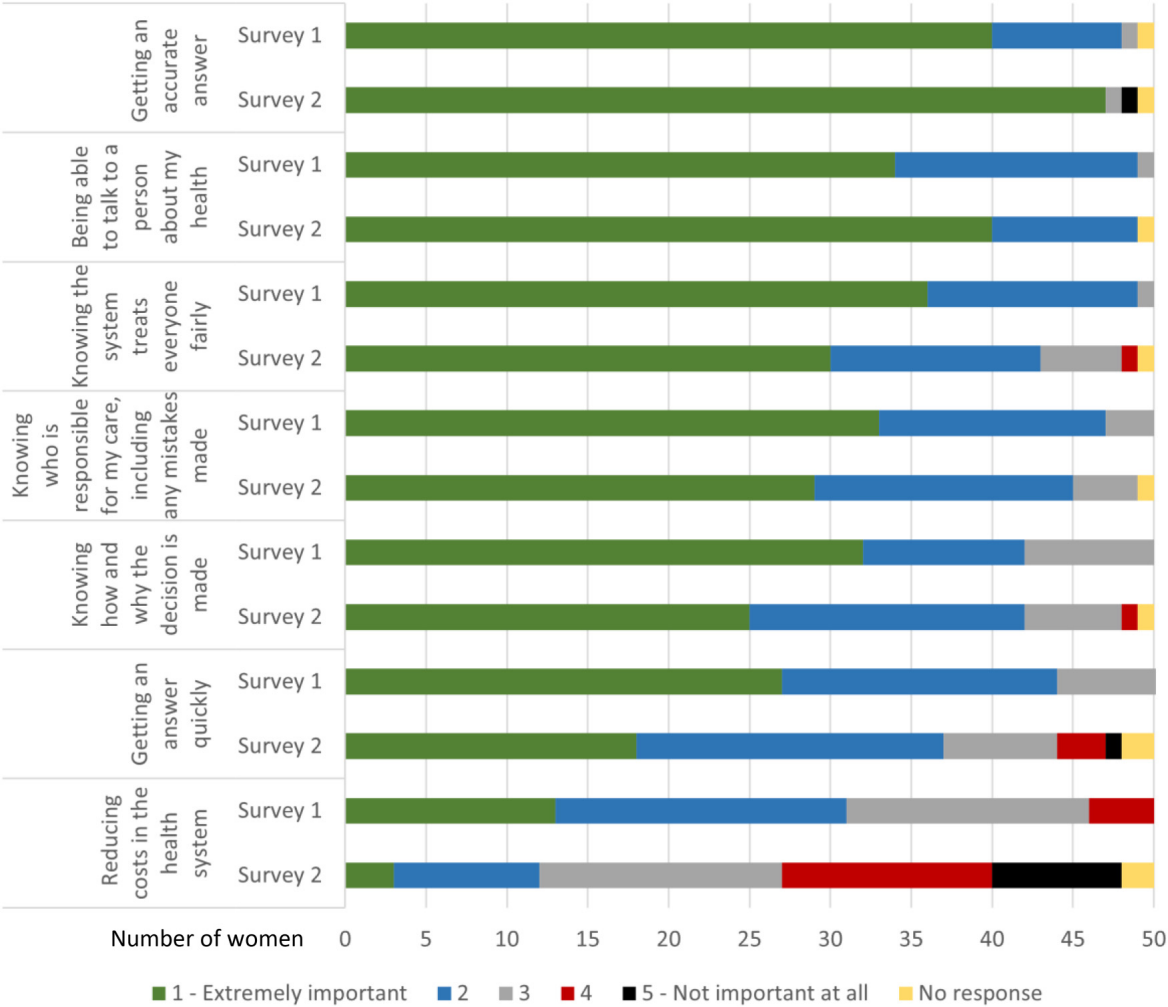
Women's individual ranking of the importance of attributes of a breast screening AI system pre-group discussion survey (Survey 1) and post-group discussion survey (Survey 2) (note small sample size).

Figure 5 presents women's rankings of the importance of different attributes of a breast screening AI system before (Survey 1) and after (Survey 2) the discussion groups. As shown, each of the seven attributes were ranked in the top 2 ‘most important categories’ by at least some of the women, before and after discussion. ‘Being able to talk to a person about my health’ and ‘getting an accurate answer’ were most important and strengthened after discussion in most groups. ‘Knowing who is responsible for my care, including any mistakes made’ and ‘knowing the system treats everyone fairly’ were also highly ranked; both softened very slightly overall, but ranking and changes in ranking across groups were varied. ‘Knowing how and why the decision is made’ increased in some groups and decreased in others, with little change overall. The importance of ‘getting an answer quickly’ was weaker and softened after discussion overall, increasing in only one group. Finally, ‘reducing costs in the health system’ was least important before discussion and softened markedly after discussion, overall and in every group. This was the only item skewed towards the ‘least important’ end of the scale.

### Qualitative findings

We present the findings in three sections to address the three research questions. Section 1 summarises women's overall responses; Section 2 highlights the two issues that mattered most in women's explanations; and Section 3 reports on women's prioritisation of values or attributes of AI in breast screening. When a quote is preceded by *[if I could talk to BreastScreen]*, it is from the end of the group discussion when women were invited to give a summing up statement: we take these to be women's most considered statements and the things they most wanted to emphasise.

### Broad—but conditional—acceptance of the use of AI in breast screening

In all eight groups, women were positive, even excited, about breast screening AI, while recognising that they had limited knowledge about AI and how breast screening currently works. Most adopted a pro-technology position. They assumed that AI would inevitably be used for social good in future, even though peoplemight experience some initial hesitancy about AI use:

I’m very positive about it. I think it can be a bit scary like anything that's new to people until it proves itself, and in five years it will have had time to do that and I think it's a really, really good idea. (DG3)

AI was expected to deliver benefits for breast screening, on implementation and increasingly over time. The main benefits women hoped for were greater accuracy—especially better cancer detection rates—and greater speed and efficiency. Women hoped these benefits would save lives, build women's confidence, and free up human staff to help breast screening keep up with population growth and demographic change. Women remembered and repeated claims from the introductory video that AI does not get bored, tired or distracted, can be trained on thousands of images quickly and can work continuously. This sometimes translated into an expectation that services would be able to screen more women more quickly, speed up internal processes, and increase access to screening:

It was cutting out the cost of a radiologist, human, and you’ve still got two opinions. So you’ve got both opinions going there to work out what's going on and that other radiologists looking with the same AI, so you’ve doubled your workforce straightaway - - -

Yes.

- - - to get more people through?

Yeah, yeah. It's ideal really. (DG8)

Participants generally believed BreastScreen, the Australian health system and health professionals could be trusted to introduce a technology only when it was safe and effective. However, residual concerns led some participants to recommend perceived safeguards, including ineffective or potentially harmful actions such as self-examination or further screening elsewhere:

But then I think our healthcare in Australia is pretty good, and implementing something like artificial intelligence, I don’t think would just be done without proper evaluation and assessment of the risks and benefits, so I wouldn’t discourage Katherine from going along for the test. Perhaps if the test was negative, I would encourage her to be very vigilant in terms of self-examination and other methods of keeping tabs on her breasts over the next two years, or even perhaps having screening somewhere else for verification. (DG6)

Although women broadly accepted AI, this was conditional in multiple ways. In the next two sections, we discuss the two most important caveats women raised.

### Human involvement and AI system performance were key to women's normative judgements

Women said two issues were keys to the acceptability of implementing AI in breast screening: the way humans and machines were combined and AI performance.

*Combining humans and machines in breast screening*. In all eight groups, women stressed that expert humans should remain as central actors in breast screening systems. Women's final message to BreastScreen often focused on this point, giving two main reasons. The first was a belief that, until AI could replicate the discernment and nuanced judgement of skilled humans, especially for atypical or borderline cases, human radiologists would be needed as a final ‘backup’ or quality assurance check. This was not simply a matter of the quantitative performance of AI vs humans; it was a qualitative matter of a perceived human intuition or ‘gut feeling’ for suspicious, subtle or unexpected changes, developed over long experience and supported by human motivation to get things right:

I guess I have had experience with doctors who just have that ability to pull things together and go, hang on a minute, maybe we need to look a little closer. And that can be really important to getting a correct diagnosis so, until an AI is at a point where it's being programmed with that nuance, I think there's still that place for that, that years of expertise. (DG8)

Most women expected that combining AI and expert human readers would produce better outcomes than using AI alone. Women were concerned that AI implementation could cause job losses, de-skilling—including loss of opportunities for human doctors to develop expert skills—job dissatisfaction and potentially machine dominance. Women argued analogically from observed job loss via automation in other sectors—for example, in supermarkets—and worried that this could be repeated in breast screening:

If the AI's going to be doing all this diagnosis, then the experts are going to have less experience and then how do we get the experienced experts because the AI's doing it?

… And then what happens if the AI fails and we don’t have the experts? (DG4)

The second reason to keep humans as central actors was to retain human contact, especially in such an intimate form of care and in the context of women's vulnerability. Some women recognised that they did not interact with the radiologists reading mammograms, so their direct experience may be unlikely to change:

I would really encourage you to not be afraid or worried about it, because I would believe that there would be less errors with AI than there would be with the human person interpreting who we never see anyway; it's not like you get to talk to someone as they’re looking at it. (DG2)

But women often emphasised the value of human contact in breast screening: physical touch, conversation, effective psychological support, the ability to ask questions about findings, avoiding alienation and retaining a sense of human connection:

I just think you need to have human intervention. Otherwise, where are we going if everything's a robot? They make cars and they do everything else and they do operations and that's fine. But you do have to talk to people, it's a very important thing for mental health more than anything else. (DG7)

Some women thought screening participants should be able to choose whether their images were read by an AI or a human or to ask for a second opinion on an AI decision.

*Very high expectations of AI performance and quality assurance*. Women put a second condition on their acceptance of breast screening AI, which was also emphasised in their closing remarks: development and quality assurance processes for AI systems, and their accuracy, must be excellent. AI systems should do better than human clinicians; some women said AI should not be implemented until it performed better than the status quo (missing fewer cancers and producing better outcomes for women):

[If I could talk to BreastScreen] I think I’d want to be telling them that accuracy is the absolute most important thing and that they should never think about bringing in something to save money or create convenience. That's the accuracy is better, at least as good, but definitely better than what's there, because that's the most important thing (DG6).

Participants had experienced computer error, mistakes and crashes and knew that humans make programming errors. Women referred to examples of spectacular computer failure, e.g. from banking, social security, autonomous vehicles, natural language processing (e.g. captioning or translation) and household smart devices. Similar failures in breast screening could result in missed cancers and deaths, so expectations on breast screening AI should be especially high:

Well, I think we’ve all seen – everybody would have seen an example of where artificial intelligence didn’t work too well. Just like Barb was saying, with the translation, for example. You’ve only got to look at the automatic captioning that happens on news reports to see that it's not infrequent to see a mistake. But that's not a very important thing but detecting breast cancer is a very important thing. So the thought that, well, it's just going to be left to some computer and it's just like, the autonomous vehicles. Nobody's confident that they are failsafe. Even though we know that humans aren’t. I mean, we know that humans … the radiologists also might miss it, but we’ve got a lot more confidence because they’ve been doing it all along. (DG5)

Because of these high stakes, women emphasised that BreastScreen should not test while implementing: a long testing period should run in parallel with service delivery—for 5, 10 or even 20 years—before full implementation. Women wanted to know how, where and by whom AI was going to be tested and reviewed and what research and evidence supported it:

[If I could talk to BreastScreen I would say] I reckon it's a really good idea, I think that I would say to them that they need to do a lot of extensive testing before it actually comes to play, because they need testing in the real world, not just – they need real testing with real women and over probably years just so that they can, people can learn to trust it and they can fix any problems that might come up that they see, which they’ll only see if they do testing in the real world. (DG3)

Women also emphasised the need for strong cross-checking and monitoring procedures and continued checking of the performance of AI systems, which could degrade over time:

[If I could talk to BreastScreen I would say] I think quality control is forever important as well. Because if the AI starts making mistakes then it might come out on other people's results as well, like it might not get picked up. So it might affect more than one person. And it's just going to save lives. So quality control is very important, and cross checking, and I think as you said before, that's probably the number one – to give you trust that it works properly. (DG1)

Women emphasised that AI performance could be better assured if humans and machines were combined: expert breast screening radiologists should cross-check AI system performance and be involved in AI development.

*Less common conditions on women's acceptance of breast screening AI*. There were some other conditions on the acceptability of breast screening AI, proposed by fewer women. In all but one group, women strongly emphasised the importance of breast screening services communicating well about AI. There was a clear expectation that BreastScreen would prepare their staff and clinicians to understand and be able to explain any AI system that was introduced and that the programme would communicate pre-emptively with women about the introduction and performance of any AI system—including providing written information, FAQs and reassurance and combatting misinformation. Some women reacted to the term ‘artificial intelligence’, finding it distracting or comical (e.g. … *every time you say artificial intelligence, I think of little … aliens running around out there, having a little confab together DG1*). Some warned against pro-innovation bias in decisions to implement AI in screening that might conflict with the values of older screening participants—even those who were relatively technologically literate:

I think sometimes people get really excited about new technologies and it's almost like a new toy thing… [but] particularly people in the older age groups haven’t grown up necessarily with this technology, and are probably a little bit more suspicious of technology, and may not necessarily … think, well just because it's a computer doing it it's definitely better; to err is human, to stuff up you need a computer. So I think they need to be aware, because it is generally older women that are, like women in our age group, that are being tested, and they’re different; we adopt technology differently to, say 20 and 30 year olds, so they need to make sure that they’re not, “Oh, we’ve got this great new toy and it's all AI and it's fantastic, and we’re getting rid of all the human stuff,” because we like humans. We like human beings and we trust them, we trust humans. (DG6)

Although never discussed at length, in half of the groups, women mentioned data safety and security and doctor–patient confidentiality. Concerns included the involvement of large tech companies such as Google and Facebook, data control, location and retention, potential identifiability from screening records and the potential for hacking and privacy breaches, including for insurance companies to be able to access the data.

Some women were concerned that deploying AI might introduce new costs to patients or that AI might be available only to those who could afford to pay. In Australia breast screening is publicly funded and free to women, so introducing AI would not introduce additional costs to Australian women. It is possible, however, that private Australian radiologists may implement AI-enabled screening before it is used in the public system, which some women may perceive as an enhanced service worth paying for.

### What will women care about most and least in breast screening AI

**Figure 6.**
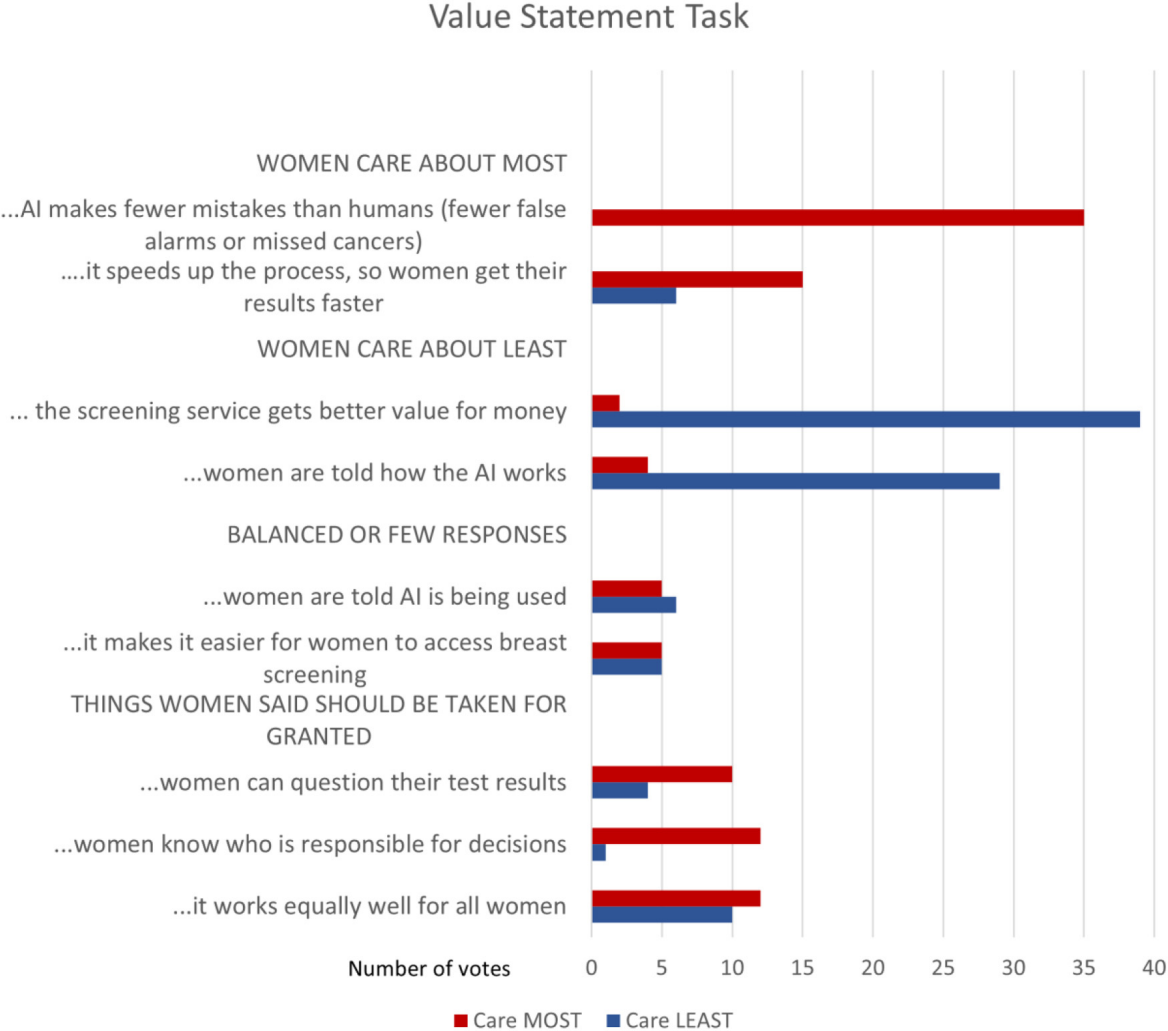
Women's responses to structured value statements (read in conjunction with qualitative analysis in text).

The findings reported above summarise key points in women's free discussion of AI's potential implementation in breast screening. In contrast, this section summarises participants’ responses to the more structured value statement task. A tally of the vote results across the eight groups is shown in Figure 6. Each woman had two ‘care about most’ and two ‘care about least’ votes, such that the total possible positive or negative vote for an item was 50.

From the nine statements, four were strongly skewed to either ‘care about most’ or ‘care about least’. We will consider the nine items in four groups (Figure 6). Note that the denominator is small, and the ranking task does not provide reliable quantitative data about women's preferences. Ranking was a prompt for discussion, and in discussion, women often qualified or interpreted their responses in ways that contradicted the prima facie rankings. We demonstrate this in the quotes and interpretation below.

### What women care about most in breast screening AI

Participants said women would care most about two things: accuracy and speed.

*The AI makes fewer mistakes than humans (fewer false alarms or missed cancers)*. Consistent with the free discussion, 35 of 50 participants (70%) said women would care most about accuracy, while none marked this ‘least cared about’ (Figure 5). Women said the purpose of breast screening was to find breast cancer: introducing a new technology that did not make this better would be nonsensical and counterproductive. Fewer mistakes also translated into fewer women experiencing poor outcomes, thus greater trust in both breast screening and AI:

Well I can’t see any point in bringing in a technology that is less accurate than human. Why – you wouldn’t do it, you would not downgrade the service that people currently have. (DG6)

*Use of AI speeds up the process, so women get their results faster*. Increased speed was a more equivocal item (Figure 5): 15 of 50 participants (30%) said women would care about it most and six least (12%). Speed was valued because waiting for such an important result was ‘agonising’, ‘nerve-racking’ and ‘stressful’; receiving a screening result resolved the tension of uncertainty, and speed could improve treatment outcomes if cancer was diagnosed. One woman noted that widespread automation was increasing shared expectations of service speed; women who lived outside cities often spoke about experiencing health service delays. The six women who de-valued speed said either that accuracy was more important than speed or that a few days delay would not make a large difference to the outcome.

### What women care about least in breast screening AI

Participants agreed that women would care least about maximising value for money and explainability (to women).

*AI provides better value for money for the screening service*. Across the groups (Figure 6), 39 of 50 participants (78%) said women would care least about AI's ability to deliver better value for money for screening services, with only two votes for the opposite viewpoint. Some women argued that health policy decisions should not be driven by financial concerns, especially for life-saving interventions, and that ‘you cannot put a price’ on serious illness like breast cancer. Some said costs were irrelevant, because Australia's screening service was already free to women and would remain so. Others recognised that costs needed to be managed but thought either that this was not important to women or that individual women should not have to think about cost (especially regarding cancer). A few women expressed concerns about private or profit-making models of breast screening service, including concern that corporate AI suppliers might absorb valuable healthcare resources.

*Women are told how the AI works*. Across the groups, 29 of 50 participants (58%) thought women would care least about explainability, with only four votes for the opposite viewpoint. Women described needing to know only that AI does work, not how it works, analogising to multiple sociotechnical systems that they use without explainability (computers, internet banking, X-rays, ultrasounds and current mammogram-reading systems). Some participants said they should know AI was being used, but information about system accuracy, and personal screening results, were more important priorities for communication with women. Explainability was seen as impractical; if women wanted to know they could investigate independently. The four participants who said women would ‘care most’ about explainability suggested this might support trust and alleviate fear of the unknown.

### Statements producing balanced or few responses

Two statements generated a small number of votes on both sides: increasing access and transparency about AI being used.

*AI makes it easier for women to access breast screening if they want to*. Only five women (10%) said access would be cared about most, reflecting a desire to widen participation; five women also said it would be cared about least, based on a belief that screening was already highly accessible. This was in part a rural–urban divide. Women from rural or remote areas explained that yes they could access screening but had to travel hundreds of kilometres and wait for weeks to get a result, so anything that might improve access was welcome. Urban women were more likely to see access as a ‘solved’ problem.

*Women are told that AI is being used*. Transparency was a common topic in the general discussion, but few participants voted on women being told AI was being used: five (10%) ‘care about most’ and six (12%) ‘care about least’. Discussion revealed a tension between the value of transparency, choice and a right to know (especially if errors are made) on the one hand and a recognition that, on the other hand, most women were willing to accept services just as they are, and automation would be increasingly ‘everyday’:

Well, I’ve just realised during the session that, for example, when we have blood tests done. Nobody tells me who's looking at the blood. And is it simply a computer using AI? Or is it a [human] technologist? And, I think, AI is everywhere today and we just have to assume it's there at all times. (DG4)

### Things women said should be able to be taken for granted

There were three items for which women's responses were especially nuanced: those on bias, responsibility and contestability. A substantial minority of women (20%–24%) chose these as their ‘care about most’ items; between 2% and 20% ‘care about least’. Across the groups, however, some women said that they had not voted for these items because they should be ‘taken for granted’, and additional some women said being limited to two values only was too difficult, so they had voted ‘not important’ for these items so they could argue that they should be a baseline expectation. Thus a ‘care about least’ or non-vote for these items may not indicate that they were unimportant to participants:

Just one other thing I would like to say though, is five, six and seven, to me, should be a given. Whether we’re using AI or with human beings. And that's the only reason I’ve discounted them. Because they should be an absolute given for any, to me, for any medical screening service.

I agree. (DG1)

*AI should work equally well for all women, no matter who they are or their background*. A total of 12 of 50 participants (24%) said women would care about this the most, and 10 of 50 (20%) the least. However in discussion, women often argued that AI should work for all women, and some participants said they voted this item down because they believed it should be already happening:

Because it doesn’t matter if you’re black or you’re white, you’re whatever colour, it's important for it to work on all women, as such. If you’re going for this screening, you’re going because you’ve got to go and it's important to have your health, so it's important to work on any background, it doesn’t matter who you are. (DG3)

A counterargument from women who voted ‘care about least’ for this item was that if the technology worked well for a subgroup of women, it should be used to benefit those women:

…if it turned out that it was enormously more effective for a particular subset of women, then it would make sense to bring it in for that subset of women, but not say everyone's got to do it, but hey, look it really works well on these particular women, so we’ll use it because it's the best technology. (DG6)

*Knowing who is responsible for decision-making*. Out of 50 participants, 12 (24%) said women would care about this item the most, and one the least; most agreed in discussion that this should be taken for granted. Women argued that responsibility was especially important in healthcare. ‘Knowing who is responsible’ could mean knowing a result (including a mistake) could be explained, accountability for errors and ensuring human review of machine decisions so final decisions were made by humans:

I think there's likely to be more questioning over a machine doing it than a person. And, I think, you want to know that there's a real person who has potentially looked at your scan at some point. (DG4)

Knowing who was responsible could increase women's acceptance and was seen as interrelated with transparency (which was presented separately in the task).

*If an AI is used to read mammograms, women can contest their results*. A total of 10 out of the 50 participants chose ‘care about most’ for this item (20%), and four ‘care about least’ (8%). In discussion women said they had a right to have their knowledge of their own bodies recognised and, to ask questions, challenge and discuss with service providers; women would have more questions if AI was introduced. A service's openness to challenge was seen as empowering women, providing peace of mind, building confidence in the system or creating a path to human review of a machine-generated result. Those women who down-voted contestability said they did not contest current results and expected things would be no different for AI-generated results. At least one participant who down-voted this item said they did so because it should be able to be taken for granted.

## Discussion

The objective of this study was to understand women's values regarding the use of AI to read mammograms in breast cancer screening; our research questions focused on women's responses to the idea of using AI in breast screening programmes (RQ1), how women explain their judgements about this (RQ2) and how women respond to the range of potentially competing values that could guide implementation of AI in breast screening (RQ3). Regarding women's overall response (RQ1), these conversations suggest Australian women are likely to accept, and be broadly positive about, the potential use of AI in breast screening. Participants’ acceptance was sometimes grounded in a pro-technology position and sometimes in a belief that technological change was inevitable. Either way, most women hoped that deploying AI might improve health outcomes for women and women's experience of breast screening.

We note, however, that women's overall responses often included two misunderstandings: to maintain trust in screening systems and ensure valid consent to the use of AI it will be important to be clear about these issues in communication about breast screening AI. First, women often imagined that mammographers (radiographers)—the professionals who take mammographic images of women's breasts—might be replaced by AI and that this would be alienating and remove essential and valued care. There is an important principle at stake here: women highly value retaining a ‘human touch’ in breast screening; consistent with this, ‘being able to talk to a person’ was ranked most important pre- and post discussion by individual women. Communication should, therefore, make clear that automation is of screen-reading of the mammograms, not image-taking, and will not change women's direct experience of being screened. Second, it made intuitive sense to women that automating screen-reading should increase access, by expanding services geographically, increasing the number of women who could be screened and/or increasing the speed of service provision. Proponents of healthcare AI often suggest that its use will increase access to healthcare. However, screen-reading is one part of a complex service with many dependencies, so it is not clear whether AI will increase access to breast screening. Communication should be clear about this uncertainty until it is resolved. Women expressed a background belief that breast screening services were trustworthy but drew on negative experiences of AI/ADM in other domains to make judgements about the likely risks of using AI in breast screening. People's experience of AI in other domains thus seems likely to shape their judgements about the use of AI in healthcare settings.

To answer RQ2, we note that women's acceptance of breast screening AI was conditional. In free discussion, the ranking task and the individual pre-/post surveys, women emphasised that AI should have excellent performance characteristics—better than current systems—*before* it was introduced. Performance as good as the status quo was not good enough, a long horizon of real-world testing was expected before implementation, and close monitoring and quality assurance were critically important. Receiving results more quickly was valued, but not at the expense of accuracy. When women had concerns about AI performance, they sometimes recommended safeguard actions—such as additional screening—that the evidence suggests would increase the risk of harm.^19^ Screen-reading AI is not currently in use in Australia, partly because we lack evidence of adequate performance and of how to deploy it in clinical workflows. Despite this, one screen-reading system has been approved for use by the Australian regulator,^20^ and others are approved in comparable jurisdictions,^21^ suggesting there may be increasing pressure to implement over time. As Hofmann has argued, health system decision-makers should be critical of the idea that new technologies must be implemented and scaled up in health systems, instead of recognising a moral imperative to deploy technologies judiciously.^22^ The centrally organised structure of public breast screening in Australia offers an opportunity to ensure rigorous evaluation,^23^ conservative decision-making about implementation and clear communication about the performance of any implemented AI systems. All of this will be needed to meet women's high expectations in the context of breast cancer screening.

The second strongly supported condition on women's acceptance was that humans must remain as central actors in breast screening, a finding consistent with the small international literature.^8–10^ Our study provides new explanations for this finding. Women wanted humans to retain control of the decision-making process with respect to both responsibility and expertise. Regarding responsibility, women's reasoning mirrored the reasoning of leading theorists of responsibility and AI:^4^ they wanted responsibility—including for final decisions—to rest with humans, thereby supporting explanation and accountability for errors. Human control also meant retaining the valued expertise of professionals who read mammograms. This expertise was perceived to include skills that machines lack, including intuitive, holistic interpretive capabilities and the ability to deal with unusual decision-making challenges; thus humans should remain as a safeguard on machine decision-making. These judgements reflect known concerns with de-skilling^24^ and with quality and safety challenges in medical machine learning.^25^ Machine learning systems are known to suffer from distributional shift, performing poorly when cases are unlike the cases used in training data and performing poorly on edge cases, often resulting in poor transportability between sites.^25^ Machines also often fail to adjust decision-making to respond to qualitative dimensions such as the relative consequences of—and thus the importance of avoiding—different kinds of error in different contexts.^25^ Women's desire to keep humans at the heart of decision-making thus should not be dismissed, as their reasons closely reflect known technical limitations of machine learning systems.

Other less common conditions included the need for good communication with staff and women about AI, the need to avoid pro-innovation bias, the need for data safety and security and doctor–patient confidentiality and the need to avoid introducing new costs to women. Together these conditions provide a context and explanation for women's support for AI in breast screening.

When asked to prioritise values (RQ3), women strongly valued accuracy, saw speed as important but valued it less after discussion, strongly de-valued explainability to women and cost-savings for health services and argued that contestability and clear lines of responsibility should be able to be assumed. Algorithmic bias has been demonstrated in medical AI systems and can exacerbate existing inequalities.^26^ Women's values regarding equity were complex: they agreed that screening programmes should be equitable, but their views on the implications for AI implementation varied. Some thought AI should not be implemented until it was universally effective; others thought it should be used in subgroups if it worked well for those subgroups. Prioritising equity will require that algorithmic systems are evaluated not just in the breast screening participant population overall but also in subgroups and that equity in performance is a target for evaluation.

### Strengths and limitations

This is one of few detailed studies with diverse women in relation to breast screening AI. Women viewed a short video and PowerPoint presentation before the discussion, so they were arguably more informed about AI than the population average. This is a challenge in research about speculative technologies, as some information inputs are needed to support a meaningful discussion. While the sample was skewed to higher levels of education, which may have impacted women's ability to engage with the content, approximately half of the participating women had not attended university, and the perspectives of these women are included in our findings. To provide a supportive environment, we likely over-represented culturally and linguistically diverse women; it is difficult to speculate on what impact this may have had on our findings. Like most consumer research on healthcare AI, women's judgements were speculative as the technology is not yet implemented in Australia. However, women drew on their experience of both breast screening and other AI-enabled technologies to make judgements and give reasons. One important limitation is that, because of the complexity of explaining screening and AI, we did not have the capacity to address the complex issue of screening outcomes and overdiagnosis: this should be addressed in future research.

## Conclusions

Women who participate in breast screening support using AI to read mammograms, but only if certain conditions are met. Key challenges for breast screening programmes are (1) to ensure that a screening system including AI performs better than the status quo; (2) to ensure human readers retain control, maintain their skills and take responsibility for outcomes; (3) to implement quality assurance that responds to women's well-founded intuitions about the weaknesses of machine learning systems; and (4) to monitor and preserve equity. Achieving this will require robust evaluation of AI systems, a conservative approach to implementation and careful communication with screening participants.

## Supplemental Material

sj-docx-1-dhj-10.1177_20552076231191057 - Supplemental material for Australian women's judgements about using artificial intelligence to read mammograms in breast cancer screening Click here for additional data file. Supplemental material, sj-docx-1-dhj-10.1177_20552076231191057 for Australian women's judgements about using artificial intelligence to read mammograms in breast cancer screening by Stacy M Carter, Lucy Carolan, Yves Saint James Aquino, Helen Frazer, Wendy A Rogers, Julie Hall, Chris Degeling, Annette Braunack-Mayer and Nehmat Houssami in DIGITAL HEALTH

sj-docx-2-dhj-10.1177_20552076231191057 - Supplemental material for Australian women's judgements about using artificial intelligence to read mammograms in breast cancer screening Click here for additional data file. Supplemental material, sj-docx-2-dhj-10.1177_20552076231191057 for Australian women's judgements about using artificial intelligence to read mammograms in breast cancer screening by Stacy M Carter, Lucy Carolan, Yves Saint James Aquino, Helen Frazer, Wendy A Rogers, Julie Hall, Chris Degeling, Annette Braunack-Mayer and Nehmat Houssami in DIGITAL HEALTH

sj-docx-3-dhj-10.1177_20552076231191057 - Supplemental material for Australian women's judgements about using artificial intelligence to read mammograms in breast cancer screening Click here for additional data file. Supplemental material, sj-docx-3-dhj-10.1177_20552076231191057 for Australian women's judgements about using artificial intelligence to read mammograms in breast cancer screening by Stacy M Carter, Lucy Carolan, Yves Saint James Aquino, Helen Frazer, Wendy A Rogers, Julie Hall, Chris Degeling, Annette Braunack-Mayer and Nehmat Houssami in DIGITAL HEALTH

sj-pptx-4-dhj-10.1177_20552076231191057 - Supplemental material for Australian women's judgements about using artificial intelligence to read mammograms in breast cancer screening Click here for additional data file. Supplemental material, sj-pptx-4-dhj-10.1177_20552076231191057 for Australian women's judgements about using artificial intelligence to read mammograms in breast cancer screening by Stacy M Carter, Lucy Carolan, Yves Saint James Aquino, Helen Frazer, Wendy A Rogers, Julie Hall, Chris Degeling, Annette Braunack-Mayer and Nehmat Houssami in DIGITAL HEALTH
