# Supplementary material for: Australian women's judgements about using artificial intelligence to read mammograms in breast cancer screening
Source: Digit Health. 2023 Aug 7;9:20552076231191057. doi: 10.1177/20552076231191057 (PMC10408316; doi:10.1177/20552076231191057)
Supplement: sj-docx-1-dhj-10.1177_20552076231191057 - Supplemental material for Australian women's judgements about using artificial intelligence to read mammograms in breast cancer screening [file sj-docx-1-dhj-10.1177_20552076231191057.docx]

# Appendix 1: Recruitment Screener Questionnaire

*So in order to assess your eligibility I need to ask you a few questions. Is that OK?*

1. Have you ever worked in breast screening or breast cancer care in the last 5 years? IF YES, RESPONDENT IS NOT ELIGIBLE: *Thank you, this study is not including people who have worked in breast screening or breast cancer care recently. Exit call.*
2. How old are you? ___
3. What is your postcode? ____
4. How long have you lived in that area?
5. What is your country of birth?
6. Do you identify with a culture other than the culture of your country of birth?
7. Are you working at the moment?
   1. Working fulltime c. Retired
   2. Working part-time / semiretired d. Unemployed
8. What did you / do you do for work? __________________
9. What is your highest qualification?
   1. School Certificate d. Undergraduate degree
   2. High School e. Postgraduate degree
   3. Trade Certificate

Caller:

*I have one last question for you, which is a little personal I’m sorry –*

1. Have you ever been diagnosed with breast cancer or DCIS? (Yes/No).

IF YES: How long ago were you diagnosed? _______________

*Caller:*

*Sorry to have to ask such a personal question – we asked because we want to be sure we are being careful about women’s experience going into the project*

**CALLER PLEASE NOTE:**

*If any women seem concerned about the question, please ask the women whether they would like information about support services and that we recognise that there may be topics raised in the dialogue groups that participants may find upsetting.*

For specific information and advice about breast screening, you can contact Breast Screen Australia on: PH: 1800 020 103,

https://www.health.gov.au/initiatives-and-programs/breastscreen-australia-program

1. Thank you. Are you happy for me to give your contact information to the research team at the University of Wollongong so they can discuss the project with you / send you some more information?

**IF NO:** Thank you very much for your time. Good bye.

**IF YES:** Thank you. Not everyone will be eligible and not everyone who is eligible will be able to be invited. The researchers will be in contact with you as soon as possible about your possible participation. What is the best way to contact you?

Phone: _____________________

Email: ___________________________

We have a package of materials to send you which you will use during the group. What is the **best address** to send these to? Your address will not be shared with the researchers or anyone else – it will only be used to send out the materials.

Expect a **call from the researchers** a few days before the group. They will explain everything that is going to happen in more detail, ask you to take a very short survey, and give you a chance to practice using Zoom if you would like that.

**Thank you and goodbye**
