# Supplementary material for: Australian women's judgements about using artificial intelligence to read mammograms in breast cancer screening
Source: Digit Health. 2023 Aug 7;9:20552076231191057. doi: 10.1177/20552076231191057 (PMC10408316; doi:10.1177/20552076231191057)
Supplement: sj-docx-2-dhj-10.1177_20552076231191057 - Supplemental material for Australian women's judgements about using artificial intelligence to read mammograms in breast cancer screening [file sj-docx-2-dhj-10.1177_20552076231191057.docx]

|  | |  | 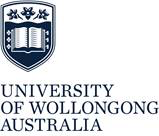 |
| --- | --- | --- | --- |
|  |  | |  |
|  |  | |  |
| **Chief Investigator:**  **Prof Stacy Carter**  **T**+61 024221 3243  **E**: [stacyc@uow.edu.au](mailto:stacyc@uow.edu.au)  **M: 0448 746 163** |  | | Room 318, Building 29  ACHEEV  School of Health and Society  University of Wollongong  NSW 2522 AUSTRALIA |

## Appendix 2: Participant Information Sheet

**Using Artificial Intelligence in Breast Screening; Dialogue Groups project**

**PARTICIPANT INFORMATION STATEMENT**

1. **What is this study about?**

You are invited to take part in a research study investigating women’s perspectives on the future use of Artificial Intelligence (AI) in mammographic breast cancer screening (breast screening).

These groups are part of larger studies examining the future use of AI in mammography in terms of its: 1) potential to early-detect 'missed' cancers; 2) cost-effectiveness; 3) ethical, legal and social implications; and 4) acceptability to women in Australia.

You have been invited to participate in this study because you are a woman who is in the age range to be eligible to participate in breast screening in Australia. This Participant Information Statement (PIS) tells you about the research study. Knowing what is involved will help you decide if you want to take part in the research. Please read this sheet carefully and ask questions about anything that you don’t understand or want to know more about.

Participation in this research study is voluntary. So it’s up to you whether you wish to take part or not. By giving your consent to take part in this study you are telling us that you:

- Understand what you have read
- Agree to take part in the research study as outlined below
- Agree to the use of your personal information as described.

1. **Who is running the study?**

This project is administered through the University of Wollongong, The Chief Investigators are:

| **Name** | **Affiliation** | **Email** |
| --- | --- | --- |
| Professor Stacy M Carter | University of Wollongong | stacyc@uow.edu.au |
| Dr Chris Degeling | University of Wollongong | degeling@uow.edu.au |
| Professor Annette Braunack-Mayer | University of Wollongong | abmayer@uow.edu.au |

The research staff are:

| Dr Yves Saint James Aquino | University of Wollongong | yaquino@uow.edu.au |
| --- | --- | --- |
| Ms Julie Hall | University of Wollongong | juliehal@uow.edu.au |
| Ms Lucy Carolan | University of Wollongong | lengland@uow.edu.au |
| Ms Tory Haywood | University of Wollongong | vhaywood@uow.edu.au |

This project is funded by the National Health and Medical Research Council (NHMRC APP1181960) and National Breast Cancer Foundation (NBCF IIRS-20-011)

1. **What will the study involve for me?**

If you agree to participate in a Dialogue Group, you will be asked to join small group online meeting (up to 7 participants) to discuss issues related to the future use of AI in breast screening. The discussion will occur on the Zoom platform. A member of our team will contact you before the discussion to record your consent to participate, ask you to complete a 5 minute survey, and provide you with any support you need to access and use Zoom.

All participants in the discussion will be women who are in the age range to participate in routine breast screening (50-74 years). During the discussion you will learn about potential uses of AI in breast screening, and will join in a discussion about alternative scenarios for the future use of AI in breast screening. You will be asked whether, in your view, those alternatives are good or bad, and why.

1. **How much of my time will the study take?**

The Dialogue Group will take about two hours.

1. **Who can take part in the study?**

Any Australian woman who is in the age range to participate in breast screening. We will be selecting participants to try and ensure that a diversity of social and cultural backgrounds, and ages, is represented in each group. Participants will need to be able to speak English.

1. **Do I have to be in the study? Can I withdraw from the study once I've started?**

Being in this study is completely voluntary and you do not have to take part. Your decision whether to participate will not affect your current or future relationship with the researchers or anyone else at the University of Wollongong.

If you decide to take part in the study and then change your mind before you participate in a discussion group, you can withdraw at any time without having to give a reason. Whatever your decision, please be assured that it will have no consequences for you. Withdrawal from the study can be organised by contacting the lead Chief Investigator Professor Stacy Carter on +61 2 4221 3243 or stacyc@uow.edu.au

If you decide to withdraw from the study, we will not collect any more information from you, and we will delete any information we have collected before the dialogue group.

Once you have participated in the discussion group, however, we will not be able to remove your contribution from the study. This is because we will be recording the discussion as a group, and will not be recording the identity of each speaker to increase confidentiality. This means we will often not be able to identify exactly who is speaking at any given time in the discussion.

1. **Are there any risks or costs associated with being in the study?**

This is a low-risk project.  The most significant risk is unwanted identification in reporting.  To minimise this risk, we will remove any details that might reveal your identity.  Digital audio files will be kept on password protected servers at all times. Aside from giving up your time, we do not expect that there will be any risks or costs associated with taking part in this study. All discussions will be moderated by an experienced facilitator to ensure that the groups are a safe and respectful place for discussion.

We acknowledge the difficulty for participants who have direct experience of breast cancer, and recognise that there may be topics raised in the dialogue groups that participants may find upsetting. We will ensure that you are supported during and after the conversation if you would like support. The group discussions will not have a focus on experiences of breast cancer: our focus will be on breast screening services, especially the possibility of using artificial intelligence in these services.

For specific information and advice about breast screening, you can contact Breast Screen Australia on:

PH: 1800 020 103, https://www.health.gov.au/initiatives-and-programs/breastscreen-australia-program

1. **Are there any benefits associated with being in the study?**

While we intend that this research study furthers understandings about attitudes to AI in Breast Cancer Screening, and develop potential policy options, it may not be of direct or immediate benefit to you. It will provide you with opportunities to contribute your thoughts and experiences which will culminate in the development of tools to guide future policy and practice.

To thank study participants and to reimburse you for some of your time, participants in the *Dialogue Groups* will be given $100 gift voucher after participating.

1. **What will happen to information about me that is collected during the study?**

If you choose to participate, this will generate:

- An audio file recording your consent* to participate
- Data from a 2-minute survey that you will take before and again after the group discussion
- A digital audio-recording of the group discussion
- A professional transcription of the group discussion

*A member of the research team will contact you prior to the research taking place to ask if you have any questions regarding this information sheet or the study process and to ask you to give a verbal consent. A sample of the verbal consent script is at the end of this document.

All the information collected from you for the study will be treated confidentially. The digital audio file will only be accessible to members of the research team. It will be kept on a password protected server.

The group discussions will be digitally recorded and transcribed to aid interpretation and analysis. Transcription of the audio files will be undertaken by a professional transcription company well known to the researchers with secure data transfer and storage facilities. The transcribers will not transcribe anyone’s names, to increase confidentiality.

The study results will be presented at conferences and in scientific publications, but we will never use your name in any of these publications or presentations. All data will be securely retained on password protected UOW servers, then destroyed 5 years after the project ceases.

By providing your consent, you are agreeing to us collecting personal information about you for the purposes of this research study. Your information will only be used for the purposes outlined in this Participant Information Statement, unless you consent otherwise.

1. **Can I tell other people about the study?**

Yes, you are welcome to tell other people about the study.

1. **What if I would like further information about the study?**

When you have read this information, Prof Stacy Carter, or another investigator on the project will be available to discuss it with you further and answer any questions you may have. If you would like to know more at any stage during the study, please feel free to contact the lead Chief Investigator Professor Stacy Carter on +61 2 4221 3243 or stacyc@uow.edu.au.

1. **Will I be told the results of the study?**

When analysis of the Dialogue Groups data is complete, researchers will provide interested participants with a summary of the findings. This will likely occur early-mid 2023. If you would like to receive information about the results, please include your email address on the Consent Form.

1. **What if I have a complaint or any concerns about the study?**

This study has been reviewed by the Social Sciences Human Research Ethics Committee at the University of Wollongong (2021/067 If you have any concerns or complaints about the way this research is conducted you can contact the Ethics Manager on (02) 4221 4457 or email [rso-ethics@uow.edu.au](mailto:rso-ethics@uow.edu.au).

1. **Sample of Verbal Consent Script**

*Hello this is [Investigator] from the Australian Centre for Health Engagement Evidence and Values at the University of Wollongong.*

*I understand that you have said that you would be willing to participate in an online group to discuss your perspectives on the use of Artificial Intelligence (AI) in breast cancer screening….*

***I would like to record your consent to participate in our project****.*

*We would normally do this face to face and collect a written consent but as it is an online group I am going to read out some details and ask if you consent.*

*I will need to audio record your consent for our records, is that ok?*

*IF* ***NO*** *THANK AND END*

*IF* ***YES*** *– turn on recorder;*

*Ok, this study has been approved by the University of Wollongong Human Research Ethics Committee.*

*Could you please state your full name: [Pause for participant to respond]*

*You were sent a copy of the Participant Information sheet, but just to remind you of a few things.*

*If you decide to change your mind about participating, you are free to withdraw at any time, up until the time of publication of findings. After publication of a paper it will not be possible to effectively withdraw your data from that analysis. Withdrawal from the study can be organised by contacting the Chief Investigator Stacy Carter, whose contact details are in the Participant Information Sheet in your participant pack.*

*The AI in Breast Screen discussion group will be audio recorded. This recording will be kept safe on the University of Wollongong’s servers, and only people directly involved in the study will have access to it.*

*Do you give your* ***consent to participate in the AI in breast screening discussion group****, to be audio recorded and for us to use the data generated under the conditions I have just described?*

*IF* ***NO -*** *That’s OK, thank you for your time. (TERMINATE CALL)*

*IF* ***YES Thank you***

*Turn off recorder?*

This information sheet is for you to keep
