# Supplementary material for: Australian women's judgements about using artificial intelligence to read mammograms in breast cancer screening
Source: Digit Health. 2023 Aug 7;9:20552076231191057. doi: 10.1177/20552076231191057 (PMC10408316; doi:10.1177/20552076231191057)
Supplement: sj-docx-3-dhj-10.1177_20552076231191057 - Supplemental material for Australian women's judgements about using artificial intelligence to read mammograms in breast cancer screening [file sj-docx-3-dhj-10.1177_20552076231191057.docx]

# Appendix 3: Survey Instrument

**University of Wollongong Artificial Intelligence Study**

Logo will be displayed at top left of screen throughout survey


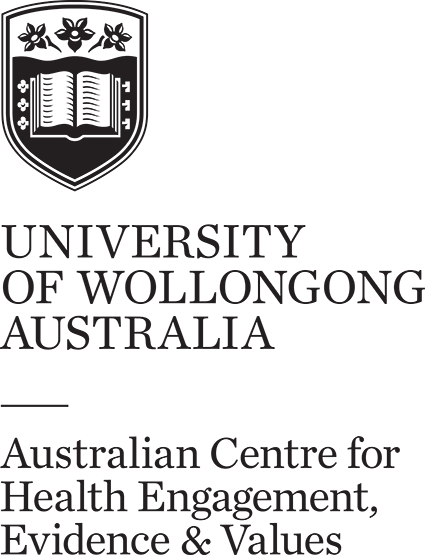


INTRO SCRIPT

*(ONLINE)

**University of Wollongong Artificial Intelligence in Breast Screen Study**

We are asking everyone participating in a discussion group about artificial intelligence in breast screening to take this short survey before and after the discussion group. You do not have to know anything about artificial intelligence to participate.

The study is being conducted by the Australian Centre for Health Engagement, Evidence and Values (ACHEEV) at the University of Wollongong (UoW).

It should take no more than 5 minutes to complete and there are no right or wrong answers. Participation is voluntary and you can withdraw at any point.

This survey includes a question about you (such as age range) and a few scenarios describing current or future uses of Artificial Intelligence (AI) in healthcare.

**If you don’t wish to answer any question, you can just click ‘Next’ to move to the next question.**

The information collected will be treated in strict confidence.

Please click the arrow tab to start the questionnaire.

A: DEMOGRAPHICS

A01 What is your age group?

1. 50-59 years

2. 60-69 years

3. 70-75 years

B: ARTIFICIAL INTELLIGENCE ANDBREASTSCREENING

*(ALL)

B01 How knowledgeable are you about artificial intelligence?

1. Not at all knowledgeable

2. A little knowledgeable

3. Somewhat knowledgeable

4. Very knowledgeable

B02 Next, we would like to ask you questions about your attitudes toward artificial intelligence.

Artificial Intelligence (AI) refers to computer systems that perform tasks or make decisions that usually require human intelligence. AI can perform these tasks or make these decisions without explicit human instructions. Today, AI has been used in the following applications:

- Translate over 100 different languages
- Predict one’s Google searches
- Block spam email
- Identify people from their photos
- Operate a robotic vacuum cleaner
- Spot abusive messages on social media
- Predict what movies or TV shows one is likely to watch online

**How much do you support or oppose the development of AI?**

1. Strongly support

2. Somewhat support

3. Neither support nor oppose

4. Somewhat oppose

5. Strongly oppose

B03 Next we would like you to consider some potential future applications of AI in the health system.

You require a medical test. Your test results are read by an AI system. The AI decides whether you have a condition, and recommends what treatments you should have.

When thinking about the health system described above, please indicate how important each of the following things are to you personally:

[DISPLAY STATEMENTS AS GRID]

a) Knowing how and why the decision is made

b) Getting an answer quickly

c) Getting an accurate answer

d) Being able to talk to a person about my health

e) Knowing who is responsible for my care, including any mistakes made

f) Reducing costs in the health system

g) Knowing that the system treats everyone fairly

[RESPONSE FRAME]

1. Extremely important

2. Very important

3. Important

4. Slightly important

5. Not at all important

B04 Finally, we would like to ask you about your attitudes toward AI in breast screening.

All Australian women aged 50-74 are invited to attend breast screening every two years. Right now in Australian breast screening programs, mammograms are read by human doctors.

However researchers are developing AI systems to read mammograms.

It is expected that these AI systems will be available for use in Australian breast screening programs within five years.

**How much do you support or oppose the use of AI to read mammograms in breast screening programs?**

1. Strongly support

2. Somewhat support

3. Neither support nor oppose

4. Somewhat oppose

5. Strongly oppose

B05 Please tell us why you support or oppose use of AI in breast screening:

____________________________________________________

____________________________________________________

____________________________________________________

____________________________________________________

____________________________________________________

CLOSING SCRIPT

Thank you for taking the time to participate. This survey was conducted by the Australian Centre for Health Engagement, Evidence and Values at the University of Wollongong.

If you would like to talk to someone about any issues that have arisen from participating in this survey, about how you have been feeling, or if you have any concerns about your mental health, please seek support from one of the services listed below:

beyondblue [www.beyondblue.org.au](http://www.beyondblue.org.au)

Phone: 1300 22 4636

Lifeline [www.lifeline.org.au](http://www.lifeline.org.au)

Phone: 13 11 14

BreastScreen <https://www.health.gov.au/initiatives-and-programs/breastscreen-australia-program>

132 050

Your answers have been submitted. You may now close the page.
