# Supplementary material for: Australian women's judgements about using artificial intelligence to read mammograms in breast cancer screening
Source: Digit Health. 2023 Aug 7;9:20552076231191057. doi: 10.1177/20552076231191057 (PMC10408316; doi:10.1177/20552076231191057)
Supplement: sj-pptx-4-dhj-10.1177_20552076231191057 - Supplemental material for Australian women's judgements about using artificial intelligence to read mammograms in breast cancer screening [file sj-pptx-4-dhj-10.1177_20552076231191057.pptx]

## Slide 1
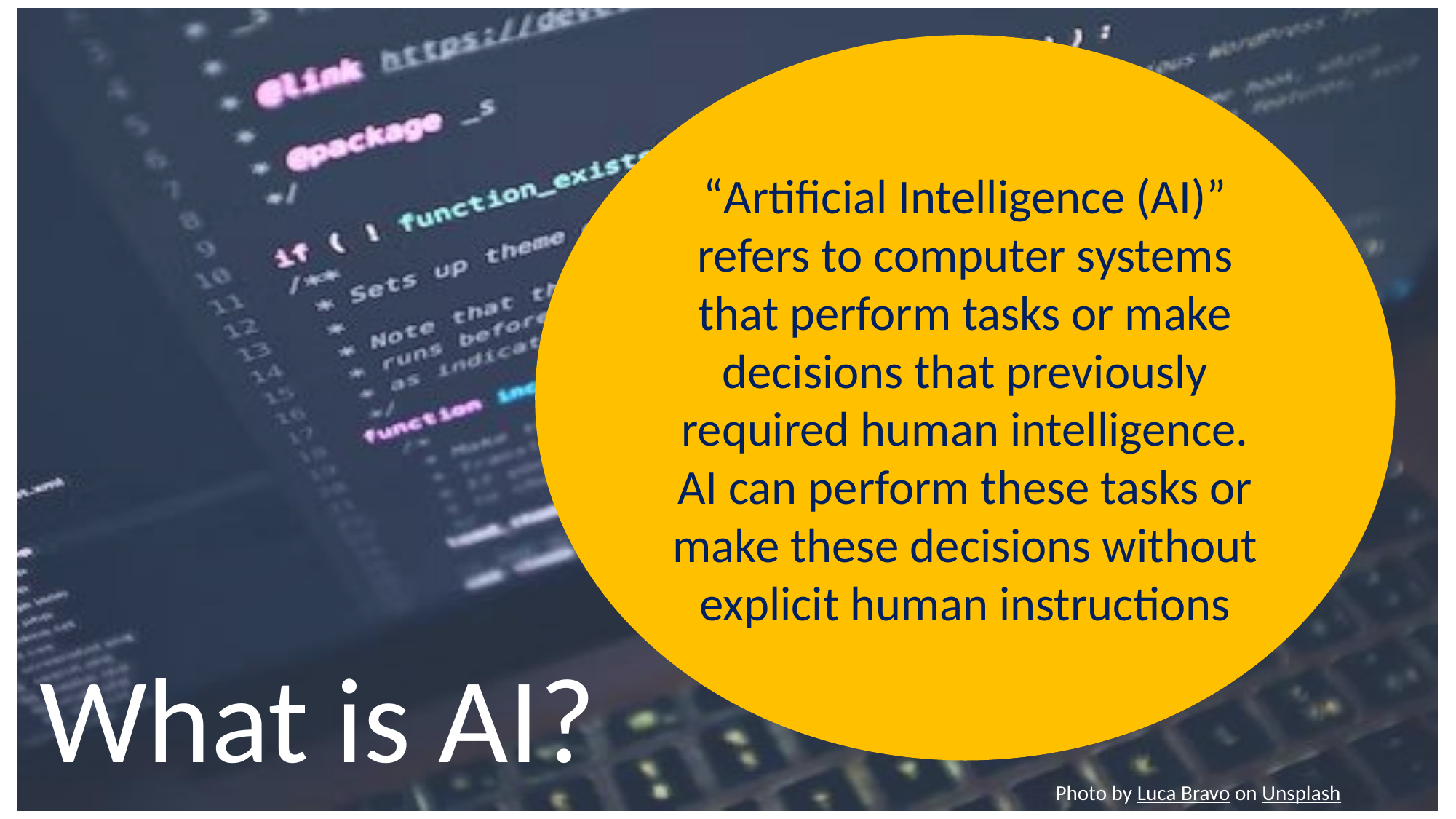

“Artificial Intelligence (AI)” refers to computer systems that perform tasks or make decisions that previously required human intelligence.
AI can perform these tasks or make these decisions without explicit human instructions
What is AI?
Photo by Luca Bravo on Unsplash

## Slide 2
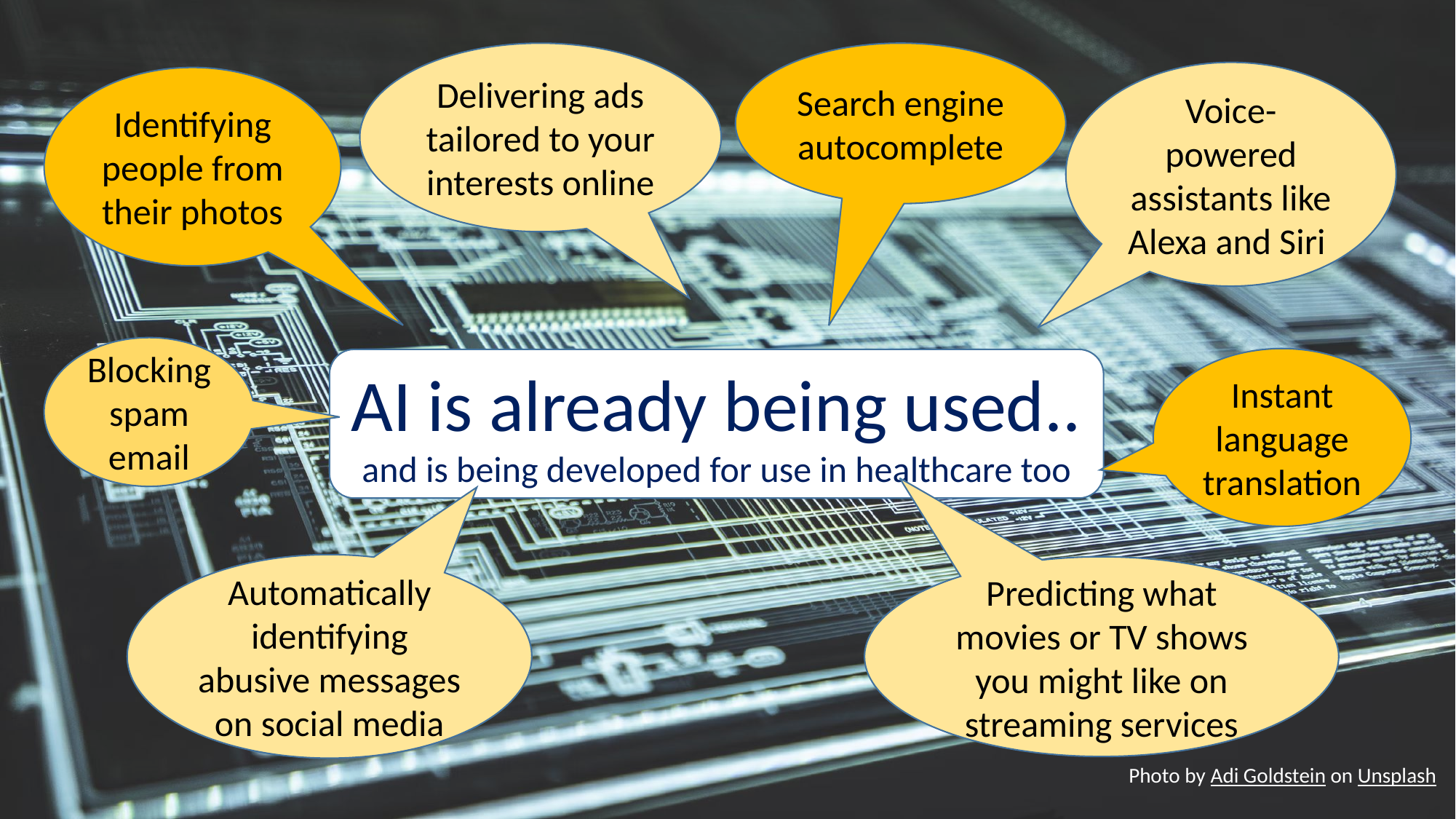

Delivering ads tailored to your interests online
Search engine autocomplete
Voice-powered assistants like Alexa and Siri
Identifying people from their photos
Blocking spam email
Instant language translation
AI is already being used..
and is being developed for use in healthcare too
Automatically identifying abusive messages on social media
Predicting what movies or TV shows you might like on streaming services
Photo by Adi Goldstein on Unsplash

## Slide 3
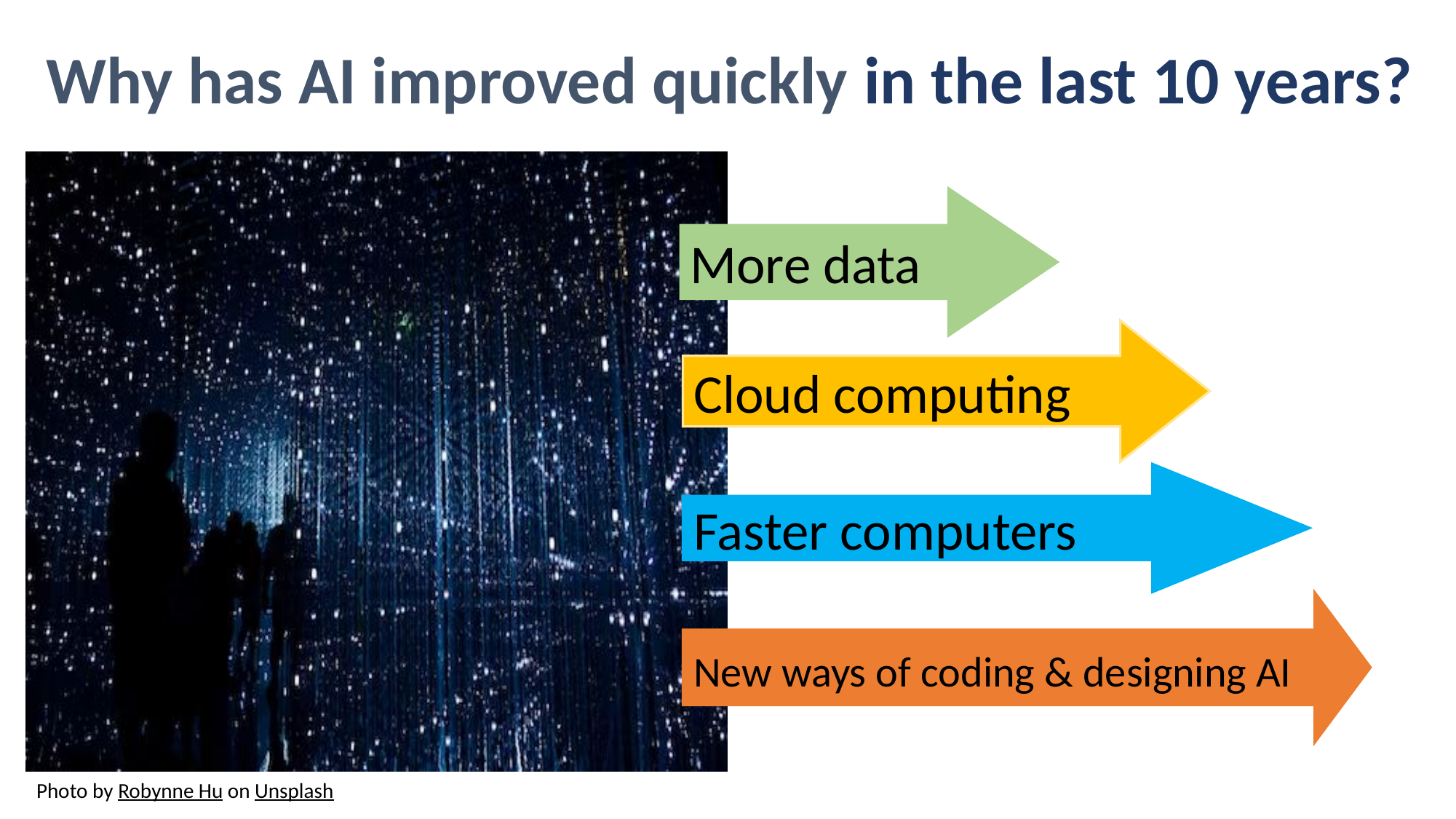

Why has AI improved quickly in the last 10 years?
More data
Cloud computing
Faster computers
New ways of coding & designing AI
Photo by Robynne Hu on Unsplash

## Slide 4
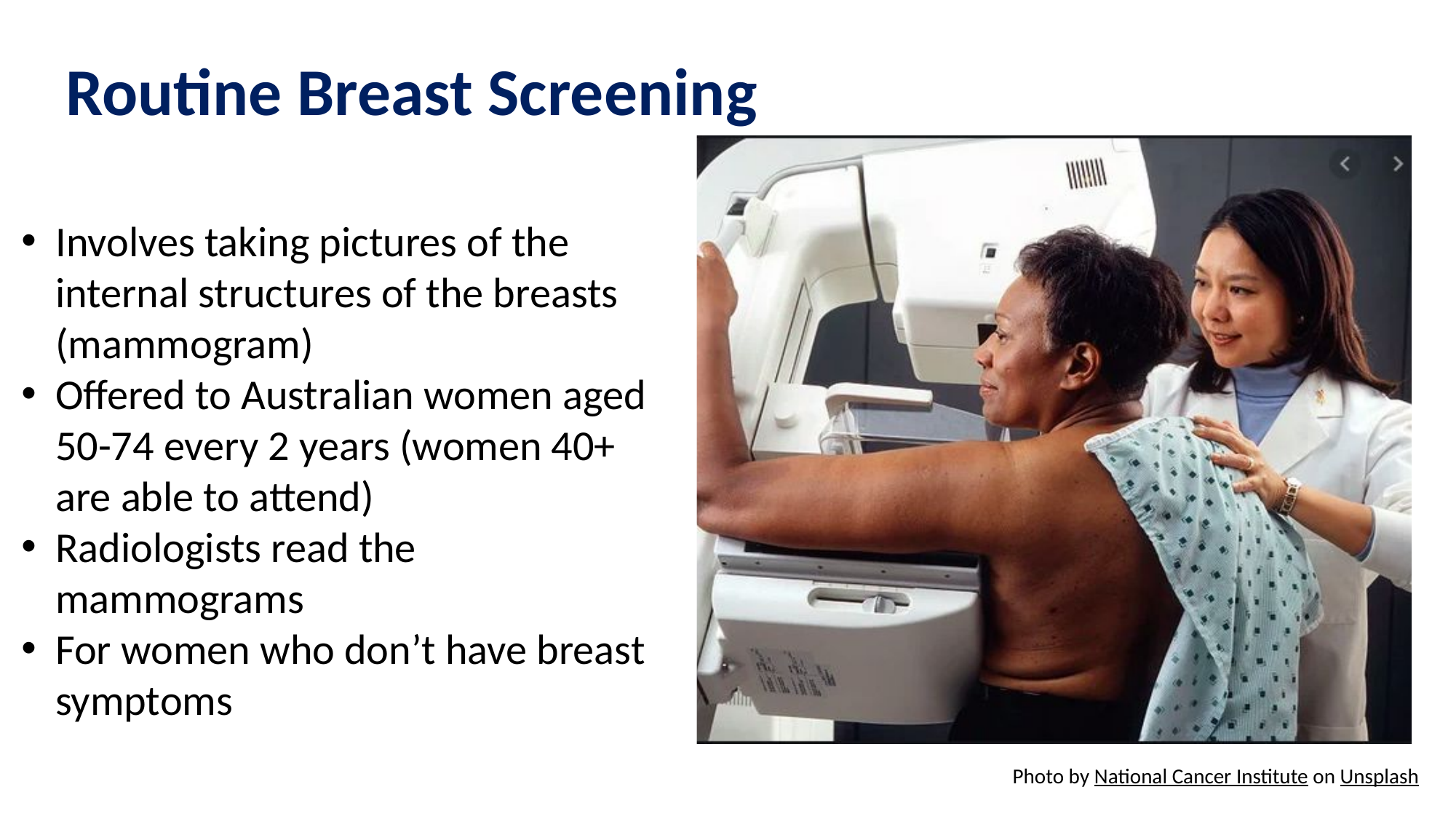

Routine Breast Screening
Involves taking pictures of the internal structures of the breasts (mammogram)
Offered to Australian women aged 50-74 every 2 years (women 40+ are able to attend)
Radiologists read the mammograms
For women who don’t have breast symptoms
Photo by National Cancer Institute on Unsplash

## Slide 5
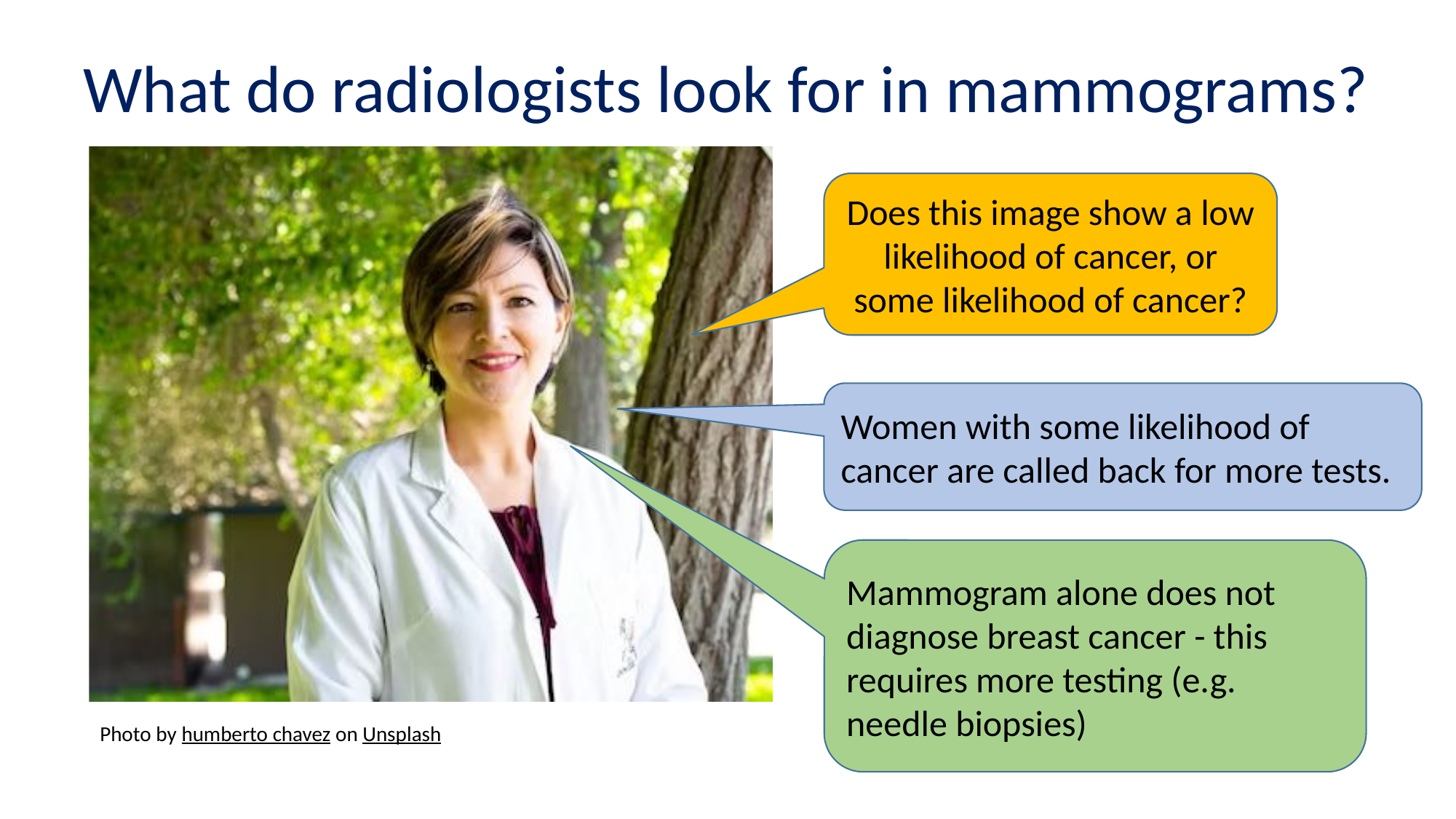

What do radiologists look for in mammograms?
Does this image show a low likelihood of cancer, or some likelihood of cancer?
Women with some likelihood of cancer are called back for more tests.
Mammogram alone does not diagnose breast cancer - this requires more testing (e.g. needle biopsies)
Photo by humberto chavez on Unsplash

## Slide 6
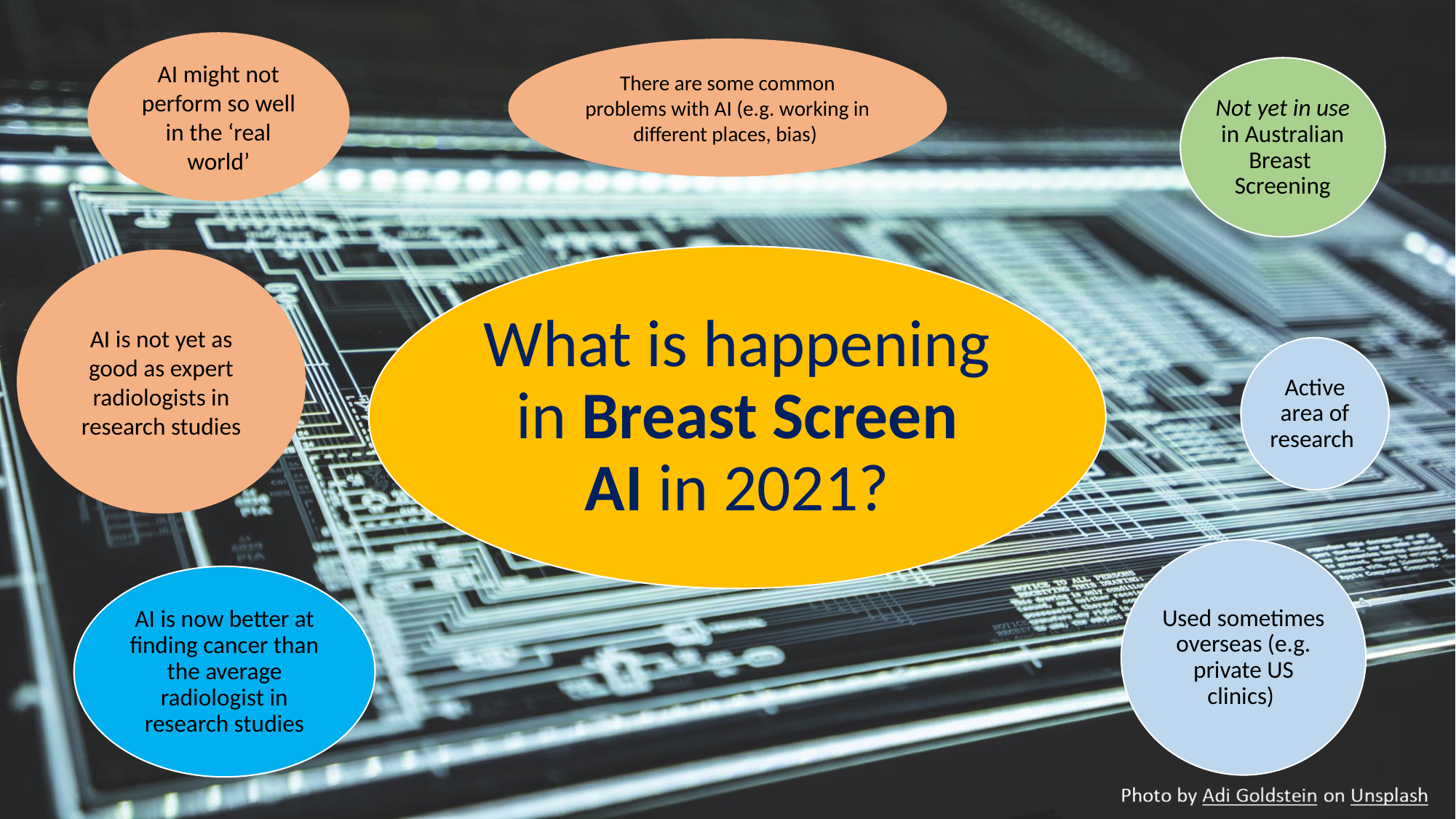

AI might not perform so well in the ‘real world’
There are some common problems with AI (e.g. working in different places, bias)
AI is not yet as good as expert radiologists in research studies

## Slide 7
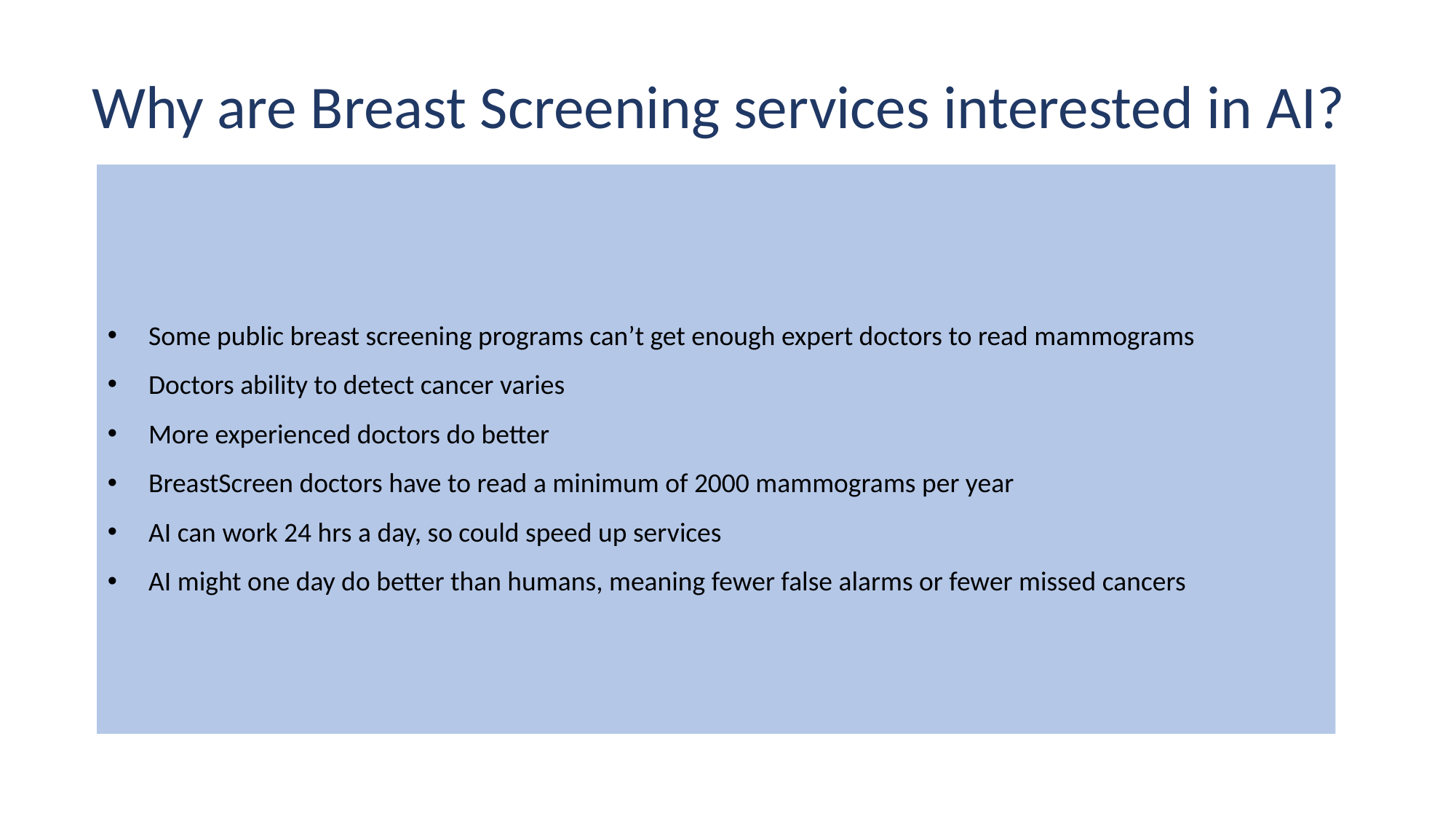

Why are Breast Screening services interested in AI?
Some public breast screening programs can’t get enough expert doctors to read mammograms
Doctors ability to detect cancer varies
More experienced doctors do better
BreastScreen doctors have to read a minimum of 2000 mammograms per year
AI can work 24 hrs a day, so could speed up services
AI might one day do better than humans, meaning fewer false alarms or fewer missed cancers
